# Two Separate Cases: Complex Chromosomal Abnormality Involving Three Chromosomes and Small Supernumerary Marker Chromosome in Patients with Impaired Reproductive Function

**DOI:** 10.3390/genes11121511

**Published:** 2020-12-17

**Authors:** Tatyana V. Karamysheva, Tatyana A. Gayner, Vladimir V. Muzyka, Konstantin E. Orishchenko, Nikolay B. Rubtsov

**Affiliations:** 1 Institute of Cytology and Genetics, The Siberian Branch of the Russian Academy of Sciences, 630090 Novosibirsk, Russia; muzyka@bionet.nsc.ru (V.V.M.); keor@bionet.nsc.ru (K.E.O.); rubt@bionet.nsc.ru (N.B.R.); 2 Institute of Chemical Biology and Fundamental Medicine, Siberian Branch of Russian Academy of Sciences, 630090 Novosibirsk, Russia; shloma@academ.org; 3 Center of New Medical Technologies, 630090 Novosibirsk, Russia; 4 Department of Genetic Technologies, Novosibirsk State University, 630090 Novosibirsk, Russia

**Keywords:** in vitro fertilization (IVF), microdissection, reciprocal translocation, small supernumerary marker chromosomes (sSMC) in humans

## Abstract

For medical genetic counseling, estimating the chance of a child being born with chromosome abnormality is crucially important. Cytogenetic diagnostics of parents with a balanced karyotype are a special case. Such chromosome rearrangements cannot be detected with comprehensive chromosome screening. In the current paper, we consider chromosome diagnostics in two cases of chromosome rearrangement in patients with balanced karyotype and provide the results of a detailed analysis of complex chromosomal rearrangement (CCR) involving three chromosomes and a small supernumerary marker chromosome (sSMC) in a patient with impaired reproductive function. The application of fluorescent in situ hybridization, microdissection, and multicolor banding allows for describing analyzed karyotypes in detail. In the case of a CCR, such as the one described here, the probability of gamete formation with a karyotype, showing a balance of chromosome regions, is extremely low. Recommendation for the family in genetic counseling should take into account the obtained result. In the case of an sSMC, it is critically important to identify the original chromosome from which the sSMC has been derived, even if the euchromatin material is absent. Finally, we present our view on the optimal strategy of identifying and describing sSMCs, namely the production of a microdissectional DNA probe from the sSMC combined with a consequent reverse painting.

## 1. Introduction

Medical genetic counseling’s key goal is to determine the probability of a child’s birth with an unbalanced karyotype in families carrying reciprocal translocations or small supernumerary marker chromosomes (sSMCs). Autosomal reciprocal translocations (ARTs) are complex chromosomal rearrangements involving two or more chromosomes. ARTs are rather frequent and lead to a chromosomal imbalance in the next generation [1]. The de novo formation or inheritance of sSMCs could also lead to genetic stability impairment [2]. sSMCs are relatively rare in the general population, found in 0.043% of newborn infants [3]. It is critical to detect impaired genetic stability and the risk of its manifestation in children carrying ARTs and sSMCs, due to the high pathogenic capacity of these mutations. Timely ART and sSMC detection might prevent a birth with an imbalance of crucial genetic regions. Parent ART or sSMC diagnosis allows them to prepare for a specific molecular cytogenetic karyotyping at the preimplantation stage and during prenatal diagnostic testing.

Currently, deletions and translocations of certain chromosomal regions are detected using commercially available DNA probes and Fluorescent In Situ Hybridization (FISH). Comparative genome hybridization on microarrays and mass parallel patient genome sequencing identifies imbalanced genomic regions with high precision. In some cases, karyomapping helps to distinctly analyze the parents’ chromosomes and define which of the parents’ chromosomes the certain chromosomal region comes from [4]. Despite significant molecular cytogenetics progress, differential staining of human metaphase chromosomes remains the most widespread and critical method of primary cytogenetic analysis. In general, any cytogenetic testing begins from the analysis of differentially stained chromosomes because the technique is simple, reliable, flexible, and cost-effective, not requiring the usage of expensive equipment and reagents. At the same time, it enables us to identify balanced chromosomal translocations and inversions, the detection of which is problematic or sometimes impossible (in cases of a preserved balance in euchromatin chromosomal regions) by many cytogenomic diagnostic methods.

A special case of chromosomal pathology is complex chromosomal rearrangement (CCR) resulting from breaks and reassociations of more than two chromosomal regions, when the possibility of the formation of gametes, with balanced euchromatin chromosome regions, is dramatically diminished; however, several studies have shown that the birth of healthy progeny is still possible [5]. At the same time, such extreme scenarios are most likely associated with abnormal meiosis [6]. Unfortunately, detailed cytogenetic diagnostics in the case of a CCR could be very problematic. Balanced CCRs could be formed either via a three-step reciprocal exchange between three chromosomes or as a result of complex translocations involving four or five chromosomes, inversions, and insertions. A vast majority of CCRs are combinations of translocations [7]. In some cases, they could lead to the formation of complex variants of rearranged chromosomes. In the current work, we provide an example of such a situation and discuss the cytogenetic testing strategy in the case of a CCR.

Another complicated case for diagnostics is sSMC [8]. In the de novo acquiring of sSMC, investigators must answer two goals: determining the origin of sSMCs and an enclosed euchromatin region’s borders. If sSMC possesses a euchromatin region that is detectable by comparative genomic hybridization, it could be simple. In case of its absence, the detection of de novo emerged sSMC requires using other techniques, such as FISH with DNA probes specific for pericentromeric heterochromatin chromosomal regions or the design of an sSMC-bearing microdissectional DNA probe with the following reverse painting on metaphase chromosomes of a patient and a healthy donor [9,10]. Notably, the elucidation of the origin of a de novo sSMC is necessary because, in 10% of cases, it is associated with uni-parental disomy (UPD) for chromosomes bearing the sSMC [11]. Notably, the prenatal cytogenetic diagnostics in identifying an sSMC in a fetus should be performed in a very narrow time window.

In the current study, we describe examples of complicated CCR and sSMC. We also discuss its diagnostics strategy and demonstrate the importance of molecular cytogenetic testing in couples with reproductive issues.

## 2. Materials and Methods

Case #1. Peripheral blood of a woman, 26 years of age. Descriptions of clinical findings: after a medical genetic diagnostic testing, no cognitive or physical abnormalities were found. She was phenotypically normal besides three underdeveloped pregnancies in anamnesis. Diagnosis—secondary infertility.

Case #2. A couple with reproductive issues: a woman, 30 years of age and a man, 35 years of age, diagnosed with primary infertility. Descriptions of clinical findings: both the male and female were otherwise phenotypically normal and there was no family history of infertility, congenital abnormalities, or mental retardation.

For both cases, sample collection was performed at the Institute of Chemical Biology and Fundamental Medicine SB RAS/the Center of new medical technologies, Novosibirsk, Russia.

### 2.1. Ethics

The studies were supervised by the Ethics Committee on Animal and Human Research of the Institute of Chemical Biology and Fundamental Medicine, Siberian Branch of the Russian Academy of Sciences, Russia (protocol #03 of 21 September 2019). Patients were enrolled in the study in strict accordance with international standards, including informed consent, acceptance of participation in the study, and confidentiality. All studies follow the ethical standards based on the Helsinki declaration of the International medical association with the year 2000 amendments. Patients involved in the study signed informed consent forms for using their respective biospecimens for scientific purposes.

### 2.2. Experimental Design

The analysis of chromosomal abnormalities consisted of several steps: (1) GTG-banding (G-bands after trypsin and Giemsa); (2) production of DNA probes via microdissection and consequent amplification of dissected material by degenerate oligonucleotide-primed polymerase chain reaction (DOP-PCR); (3) FISH of microdissection DNA probes on patients’ metaphase chromosomes. If a DNA probe is derived from an abnormal chromosome, the FISH is performed on metaphase chromosomes of both the patient and a healthy volunteer. FISH with a loci-specific DNA probe was used to identify regions in rearranged chromosomes (Figure S1).

### 2.3. Metaphase Chromosome Sample and Slide Preparation

Lymphocytes from peripheral blood were cultivated according to the standard techniques [12]. Slides with metaphase and prometaphase chromosomes for FISH were prepared as previously described [13].

GTG differential staining of metaphase chromosomes was performed according to the standard protocol. Differentially stained chromosomes were analyzed using light microscope OLYMPUS CX41 (Tokyo, Japan). A CCD camera and VideoTesT-Karyo 3.1 software (Ista-Video Test, Saint-Petersburg, Russia) were used for the image acquisition.

### 2.4. FISH DNA Probes Preparation

Microdissectional whole chromosome painting DNA probes for chromosomes 3, 5, 14, 15, and 16; partial chromosome painting DNA probes for the short and the long arms of chromosome 16 and certain segments of chromosomes 3 and 14 were amplified from 5–10 copies of respective chromosomes or chromosomal regions, as described previously [14,15]. DNA probes derived from rearranged chromosomes or sSMC were amplified from one copy using the same protocol. The labeling of DNA derived from microdissectional DNA libraries was performed with 17 additional cycles of degenerate-oligonucleotide-primed polymerase chain reaction (DOP-PCR) with AlexaFluor 488-5-dUTP (MolecularProbes, Eugene, OH, USA) or TAMRA-5-dUTP (5-Tetramethylrhodamine-dUTP, MolecularProbes, Eugene, OH, USA) [16]. Three-color FISH microdissectional DNA libraries were labeled with Alexa-488 dUTP, 5-TAMRA-dUTP, and Cy5-dUTP in 17 additional DOP-PCR cycles. The quality of all DNA probes prepared with chromosome microdissection was tested by Chromosomal in Situ Suppression (CISS) hybridization on metaphase chromosomes of a donor with normal karyotype.

FISH of microdissectional DNA probes on metaphase chromosomes of a healthy donor or patient was performed according to a standard protocol of CISS hybridization with suppression of hybridization of repetitive sequences [17]. The general staining of chromosomes was performed using 4’,6-Diamidino-2-phenylindole dihydrochloride (DAPI) (Sigma, Darmstadt, Germany). Chromosomes and chromosomal regions were identified by analyzing inverted DAPI banding. Karyotypes are reflected according to the international cytogenetic nomenclature [18].

Alu-DNA probe was produced as described [19]. FISH of the Alu-DNA probe was performed without suppression of repetitive sequences hybridization. The technique allowed us to stain euchromatin chromosomal regions, but no heterochromatin regions. FISH with this DNA probe detected a small region with Alu repeats in the satellites of human acrocentric chromosomes [19].

The DNA probe used for visualization of 18S ribosomal DNA (rDNA probe) was a 3.2 kb fragment of human 18S rDNA in pHr13 [20,21]. It was labeled with TAMRA 5-dUTP by nick translation. To detect telomeric clusters, the (TTAGGG)n probe labeled with TAMRA 5-dUTP was used [22].

After performing FISH, chromosomes were stained with DAPI. Microscopic analysis after FISH was performed on AxioPlan 2 Imaging microscope (Zeiss, Jena, Germany) with the filters kit #49 (Zeiss, Germany), SP101 FITC (CHROMA, Wixom, MI, USA), and SP103v1 Cy3tmv1 (CHROMA, Wixom, MI, USA), CCD camera (CV M300, JAI Corporation, Miyazaki, Japan). For signal registration, ISIS5 software (METASystems GmbH, Altlussheim, Germany) was used. All microscopy experiments were performed at the Core Facility for microscopic analysis of biological objects at the Institute of Cytology and Genetics SB RAS, Russia.

## 3. Results

**Figure 1:**
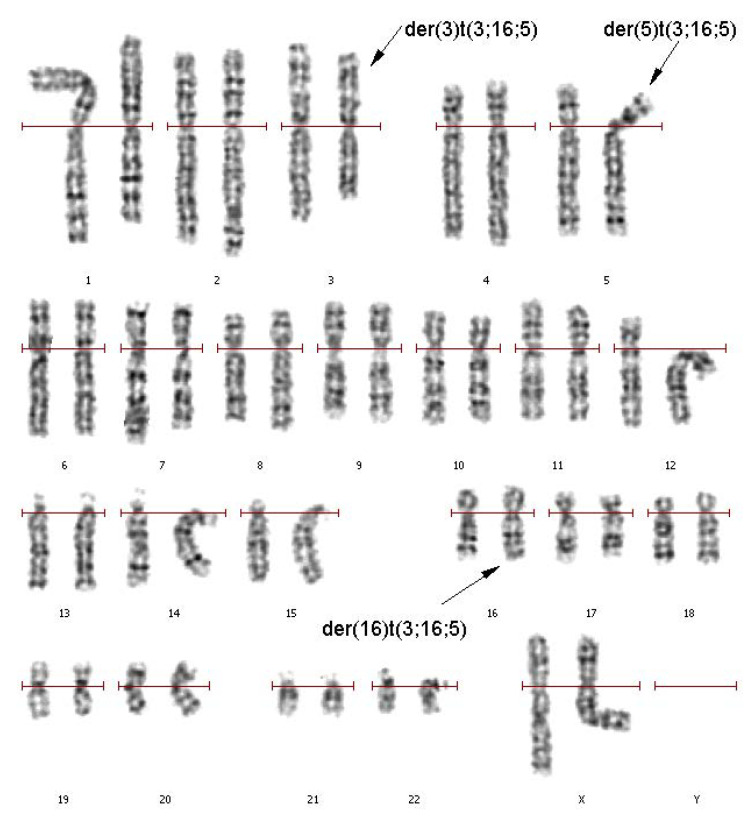
GTG (G-bands after trypsin and Giemsa) differentially stained chromosomes of a patient with a complex chromosomal rearrangement (CCR). der(3)t(3;16;5), der(5)t(3;16;5), der(16)t(3;16;5) reflect derivatives of chromosomes 3, 5, and 16 involved in the CCR.

A primary cytogenetic analysis was performed on the 72-h lymphocyte culture derived from the patient’s peripheral blood. GTG differential staining of metaphase chromosomes revealed a balanced translocation involving chromosomes 3, 5, and 16. Patient’s karyotype was described as 46,XX,t(3;16;5)(q25;q21;p15.1) (Figure 1).

**Figure 2:**
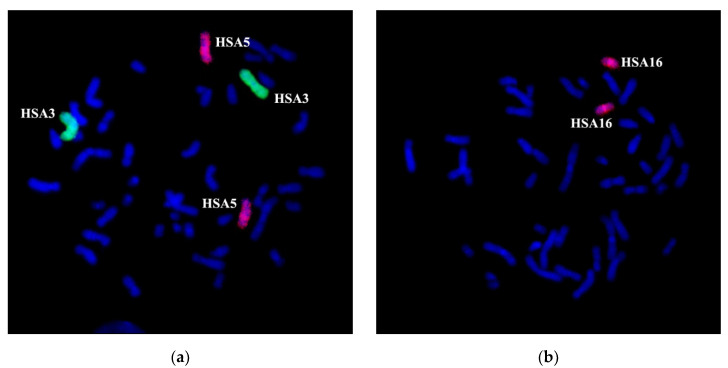
Two-color chromosomal in situ suppression (CISS) hybridization with WCP3 (green)/WCP5 (red) (**a**) and WCP16 (red) (**b**) on metaphase chromosomes of a healthy donor. Whole chromosome painting probes (WCPs) intensely painted original chromosomes. The general staining of chromosomes with 4’,6-Diamidino-2-phenylindole dihydrochloride (DAPI) in blue. Chromosomes are marked as human chromosome 3 (HSA3), human chromosome 5 (HSA5), and human chromosome 16 (HSA16).

For reassuring the correctness of conclusions made during the analysis of GTG differentially stained chromosomes, we performed additional cytogenetic testing with WCP3, WCP5, and WCP16, which stain the respective chromosomes. Whole chromosome painting probes (WCPs) were obtained by microdissection of metaphase chromosomes with the following DOP-PCR of the isolated chromosomal material. The quality of produced DNA probes was checked by CISS hybridization on metaphase chromosomes of a healthy donor (Figure 2). Obtained microdissected WCPs intensively painted original chromosomes showing their suitability for the analysis of rearranged chromosomes. A specific signal was detected on the chromosomes homologous to the prepared DNA probes; therefore, microdissectional DNA probes could be used for the analysis of chromosomal abnormalities.

**Figure 3:**
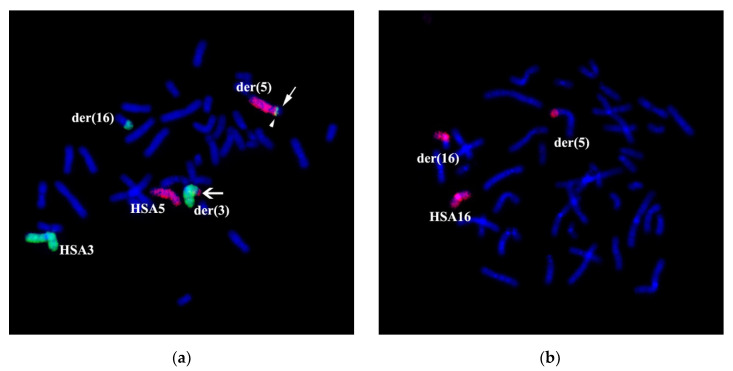
Chromosome painting of patient’s chromosomes. Two-color CISS hybridization on the patient’s metaphase chromosomes with DNA probes WCP3 (green)/WCP5 (red) (**a**) and WCP16 (red, (**b**)). DAPI staining is in blue. Intact complete chromosomes are indicated as HSA3, HSA5, and HSA16. (**a**) →—arrow shows the absence of a signal on the short arm of the chromosome 5 derivative. ▶—arrowhead indicates the WCP3 signal (green) on the short arm of the chromosome 5 derivative. ➔—arrow indicates WCP5 (red) signal at the distal region of the long arm of chromosome 3. (**a**) Chromosome 3 material was detected on chromosomes der(5) and der(16). (**b**) Chromosome 16 material was detected on the short arm of chromosome der(5).

Two-color FISH with WCP3 and WCP5 on the patient’s metaphase chromosomes specifically labeled chromosomes 3 and 5, as well as regions of these chromosomes into rearranged chromosomes. According to the patient’s karyotype description based on the analysis of GTG differential staining, WCP3 stained a portion of der(3) chromosome and a distal region of the long arm of der(16) chromosome in addition to an intact chromosome 3 (Figure 3a). WCP5 stained an intact chromosome 5, 5p15.1→qter portion of der(5) chromosome and a distal region of the long arm of der(3) chromosome (Figure 3a). WCP16 labeled an intact chromosome 16, the short arm and a small proximal region of the long arm of der(16) chromosome, as well as a distal region of the short arm of the der(5) chromosome (Figure 3b). Besides the staining pattern, which is expected after an analysis of GTG differential chromosomal staining, WCP3 labeled a small region of chromosome 3 at the short arm of the der(5) chromosome (Figure 3a). A portion of the HSA3 chromosome on the der(5) chromosome does not match the patients’ karyotype description based on the analysis of GTG differentially stained chromosomes. The results of the WCP3 painting suggest that, during the reorganization of the HSA3 chromosome, it was cleaved at least in two distinct regions, breaking it into three parts. The identification of the chromosome 3 region, re-located to chromosome der(5), and the confirmation of the contents of the translocated chromosomal regions of chromosomes, required additional experiments.

**Figure 4:**
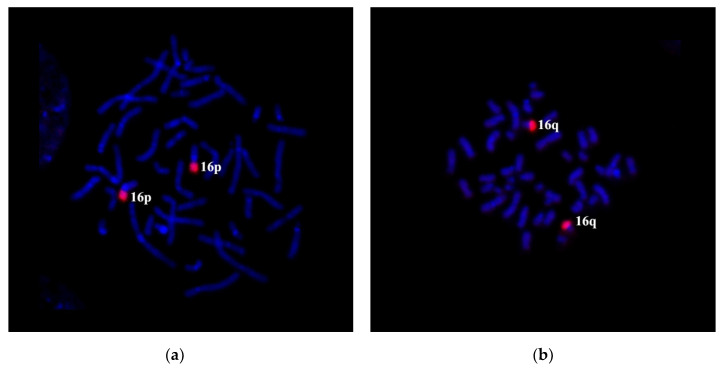
Quality control for microdissection DNA probes homologous for short and long arms of chromosome 16. CISS-hybridization of DNA probes PCP16p (**a**) and PCP16q (**b**) on metaphase chromosomes of a healthy donor. DAPI staining is in blue. Both 16p and 16q indicate the short and the long arm of chromosome 16, respectively.

We amplified microdissectional DNA probes from the short arm and C-negative part of the long arm of chromosome 16 to confirm the translocation of 16q21→qter region to chromosome 5. The specificity of DNA probes was tested on metaphase chromosomes of a healthy donor. Partial chromosome paints: PCP16p and PCP16q probes labeled C-negative regions of the respective arms of chromosome 16 (Figure 4a,b).

The intensity at the proximal portions of stained regions was slightly diminished. Perhaps the number of assembled copies of proximal edge regions was decreased due to the ambiguity of identifying C-negative and C-positive region borders and excluding constitutive heterochromatin away from the dissection sample.

**Figure 5:**
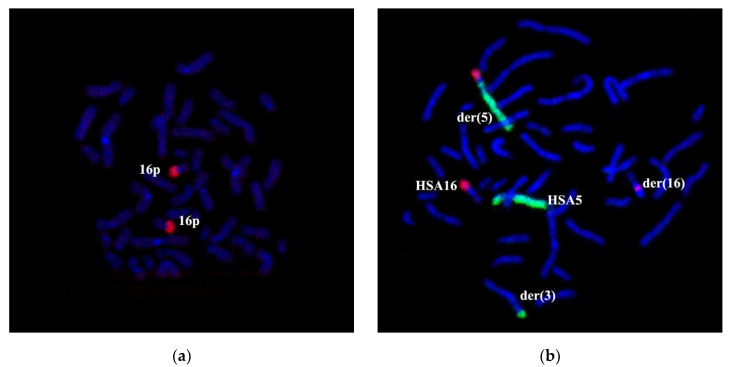
CISS hybridization with PCP16p (**a**) and WCP5 (green)/PCP16q(red) (**b**) on the patient’s metaphase chromosomes. (**a**) PCP16p DNA probe that is homologous to the short arm of chromosome 16 (red). Chromosomal regions where the specific signal was detected are indicated: 16p—short arms of chromosomes 16 and chromosome 16 derivative. (**b**) Chromosomal regions where the specific signal was detected are indicated: HSA5, HSA16—intact chromosomes 5, 16. der(3), der(5), der(16)—chromosomes 3, 5, and 16 respective derivatives. Chromatin DAPI staining is in blue.

PCP16p and PCP16q stained the patient’s chromosomes according to GTG differential staining results and the respective suggested patient’s karyotype. PCP16p labeled the short arms of chromosomes 16 and der(16) (Figure 5a). PCP16q stained the long arm of chromosome 16, a small proximal region of the long arm of chromosome der(16) and a distal portion of the short arm of chromosome der(5) (Figure 5b). This staining pattern resembles the initial karyotype description (Figure 1). The low intensity of the 16q12.1→q21 region staining in chromosome der(16) could be explained by both decreased intensity of a proximal C-negative part of 16q and spreading of preserved DNA of 16q12.1→q21 region in chromosome der(16) into neighboring chromosomal bands.

**Figure 6:**
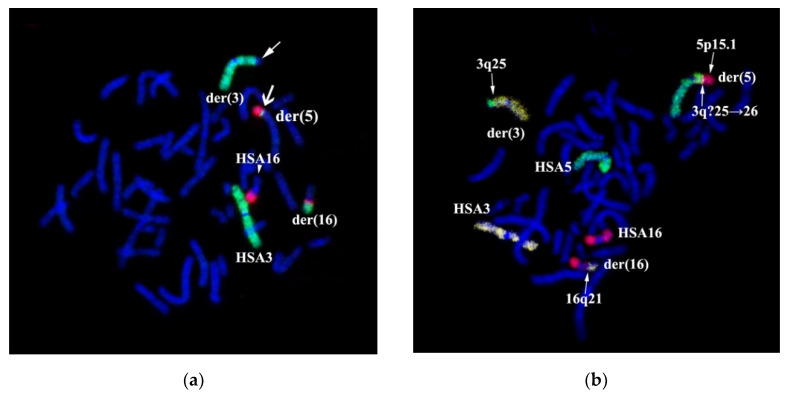
Two-color WCP3(green)/PCP16q(red) (**a**) and three-color WCP3(yellow)/WCP5(green)/WCP16(red) (**b**) CISS-hybridization on the patient’s metaphase chromosomes. (**a**) ➔—arrow indicates WCP3 (green) signal at the short arm of chromosome 5 derivative. →—arrow indicates the absence of a signal at the long arm of chromosome 3 derivative. Three-color CISS hybridization WCP3(green)/WCP5(green)/WCP16(red) (**b**) on metaphase chromosomes of the patient. Chromosomes and chromosomal regions with specific signals are indicated. (**b**) →—arrow indicates breaking points in 3q25; 16q21; 5p15.1 loci. Further, 3q?25→26 is a chromosome 3 segment detected by fluorescent in situ hybridization (FISH) in the short arm of chromosome 5 derivative. CISS hybridization revealed a region from chromosome 3 in der(5). Chromatin DAPI staining is in blue.

Three-color FISH with WCP3, WCP5, and WCP16 probes demonstrated that the chromosome 3 segment, supposedly 3q?25→26, is located between regions derived from chromosomes 5 and 16 (Figure 6b). CISS hybridization results with WCP3/WCP5/WCP16 probes on the patient’s chromosomes resemble GTG chromosomal labeling results, excluding the insertion of chromosome HSA3 material into chromosome der(5) to be localized between a sub-band 5p15.1 and a band 16q21. To determine the localization of this insertion, we amplified a microdissectional DNA probe from chromosome der(5).

**Figure 7:**
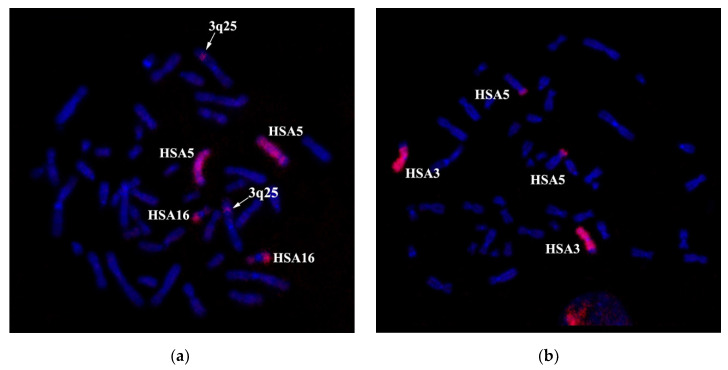
CISS-hybridization of microdissection DNA probes prepared from single abnormal chromosomes: PCPder(5) (**a**) and PCPder(3) (**b**) DNA probes on metaphase chromosomes of a healthy donor. Chromosomes 3, 5, and 16 with a specific red signal are indicated. Chromatin DAPI staining is in blue.

CISS-hybridization with PCPder(5) labeled 5p15.1→qter, 16q21→qter and 3q25 (Figure 7a). CISS-hybridization PCPder(3) stained 3pter→3q25 and 5p15.1→5pter (Figure 7b).

Based on our results, we cannot conclude the presence or the absence of 3q25 region duplication in the patient’s genome. For the completion of the analysis, we made a DNA probe from the der(5) chromosome (PCPder(5)). In the case of a duplication of the material of the 3q25 band, one would expect that CISS hybridization with this DNA probe would label a region in chromosome der(3) besides chromosome der(5), 16q21→qter region in the intact chromosome 16 and 3q25 band in the intact chromosome 3; however, PCPder(5) stained only the regions mentioned above (Figure 7a).

**Figure 8:**
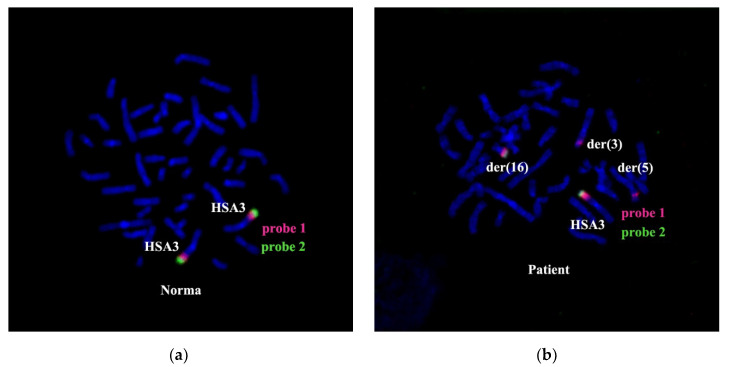
CISS hybridization with PCP3q25→q26 (probe1) and PCP3q25→qter (probe 2) on chromosomes of a healthy individual (**a**) and the patient (**b**). Chromosomes with signals are indicated: HSA3—intact chromosome 3, der(3), der(5), and der(16)—derivatives of chromosomes 3, 5, and 16, respectively.

The combined in situ hybridization results with two different region-specific microdissectional DNA probes confirmed the presence of 3q25 band in chromosome der(5). Probe 1 was produced from 3q25→q26 ((PCP3q25→q26), and probe 2 from 3q26→qter (PCP3q25→qter). Both probes label the respective chromosome 3 regions (Figure 8).

**Figure 9:**
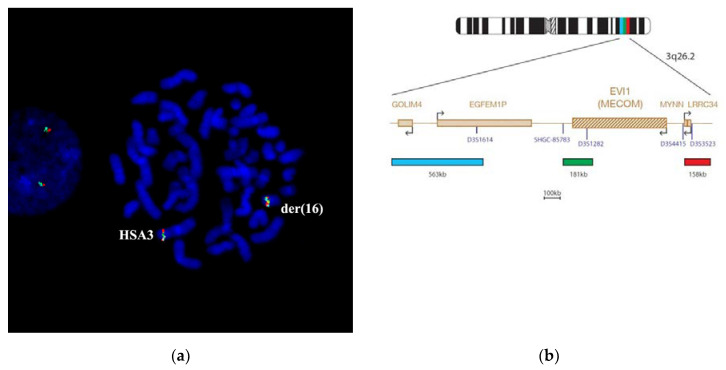
The analysis of 3q26.2 chromosomal segment. (**a**) FISH EVI1 Breakapart Probe on the patient’s chromosomes. (**b**) Scheme of EVI1 Breakapart Probe homology. The EVI1 3q26.2 product consists of a 158 kb probe, labeled in red, telomeric to the D3S4415 marker and including the LRRC34 gene, a green probe covering a 181 kb region, including the entire *EVI1* gene and flanking regions and a blue probe, which covers the 563 kb region centromeric to the *EVI1* gene, including the D3S3364 marker. Signal was detected at chromosomes 3 and der(16). Chromatin DAPI staining is in blue.

After CISS hybridization on the patient’s chromosomes PCP3q25→q26 and PCP3q25→qter, besides the standard pattern of painting on intact chromosome 3, we identified labeled regions on the rearranged chromosomes der(3), der(5), and der(16) (Figure 8). The painting pattern for der(16) chromosome region was like the pattern of 3q25→qter; however, a region labeled with PCP3q25→ q26 on der(16) chromosome was of a smaller size. PCP3q25→ q26 also stained small portions of der(3) and der(5) chromosomes. Results of FISH with DNA probe EVI1 Breakapart Probe (https://www.cytocell.com/probes/27-evi1-mecom-breakapart) (fig EVI1), which labels 3q26.2 (Figure 9) also match the conclusion that the insertion of chromosome 3 material into der(5) chromosome resembles band 3q25. A specific signal was detected only in 3q26.2 of an intact chromosome 3 and a distal region of chromosome der(16). All this suggests that the patient’s karyotype has undergone two consequent translocations.

The obtained results confirm the notion that the patient’s karyotype forms after two consequent translocations. The was, probably, the reciprocal translocation t(3;5)(q25;p15.1), and then the exchange between 3q25→3qter and 16q21→16qter regions; however, a breakpoint in the 3q25 band appeared to be localized more distally to the breakpoint for the first translocation. As a result of this shift, a small region of chromosome 3 was preserved between the material of chromosomes 5 and 16; therefore, despite a complex chromosome reorganization, a balanced genotype is preserved. Using the comparative genomic hybridization (CGH) microarray or even full genome sequencing, one could miss the abnormalities described in the above section. Simultaneously, the FISH analysis confirmed the presence of the reciprocal translocation involving three chromosomes (3, 5, and 16) and enabled us to detect the insertion of a small additional portion of chromosome 3 at the short arm of chromosome 5 derivative.

The molecular cytogenetic analysis enabled us to clarify the organization of der(5) chromosome and to describe the patient’s karyotype 46,XX,der(3)(3pter→3q25::5p15.1→5pter),der(5)(16qter→16q21::3q25::5p15.1→5qter),der(16)(16pter→16q21::3q25→3qter).

### Small Supernumerary Marker Chromosome (sSMC)

**Figure 10:**
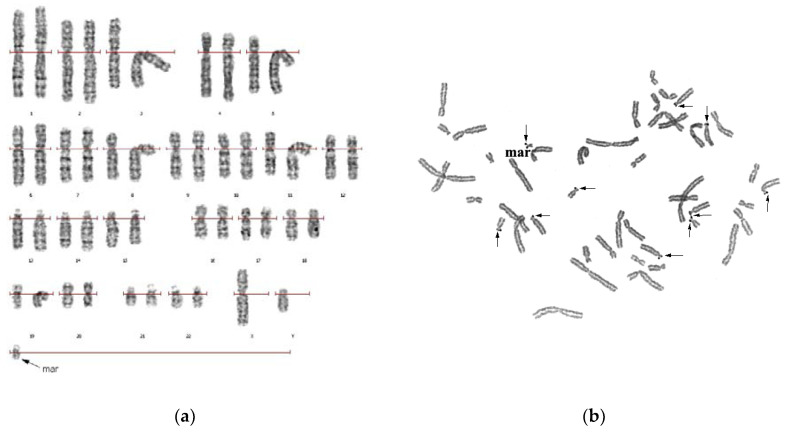
The karyotype of the patient. (**a**) GTG-differentially stained chromosomes of the patient with small supernumerary marker chromosome (sSMC) in the karyotype. (**b**) Metaphase plate of a patient with AgNOR-staining (case 2). →—arrow indicates nucleolus organizer region (NOR) detected on acrocentric chromosomes and sSMC on the patient’s sSMC by AgNOR-staining. mar—indicates sSMC.

Karyotyping of a couple with reproduction issues by analyzing GTG-differentially stained chromosomes can detect an sSMC in all 15 karyotyped cells of a man. Any other structural or numeral abnormalities were not detected. The patient’s karyotype (Figure 10) was described as 47,XY,+mar. In one of the arms of the sSMC, a secondary construct and a satellite could be seen. Ag-NOR differential staining has detected an active nucleolus organizing region (NOR) (Figure 10b), suggesting that sSMC is derived from one of the acrocentric chromosomes.

**Figure 11:**
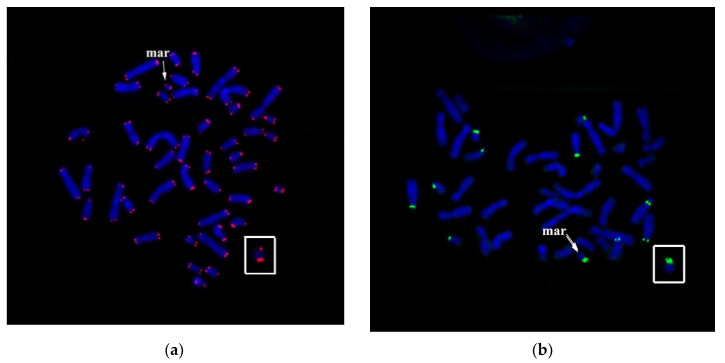
FISH with Tel-DNA probe (**a**) and rDNA-probe (**b**) on the patient’s metaphase chromosomes. mar and arrows indicate sSMC.

Interestingly, most of the sSMCs derived from acrocentric chromosomes are inverted duplications of the short and a part of the initial chromosome’s long arm. The lack of nucleolus organizer region on the second arm suggests that sSMC was formed after deleting a big part of an acrocentric chromosome’s long arm. Alternatively, it is the result of translocation. Previously, sSMCs, which include a proximal region of the initial chromosomes and other regions, have been described [23,24,25,26,27]. In some cases, Ag-NOR staining detects regions other than the nucleolus organizer region. FISH with labeled human rDNA (rDNA probe) or labeled human DNA of a telomere repeat (TTAGGG)n. Tel-DNA probe (Figure 11a,b) was performed to confirm the presence and the stability of nucleolus organizer region within sSMC.

Clusters of ribosomal DNA were detected on the short arms of five pairs of acrocentric chromosomes (HSA13, HSA14, HSA15, HSA21 and HSA22), and one of the arms of the sSMC (Figure 11b). FISH with Tel-DNA probe demonstrated the presence of clusters of telomeric repeats at the ends of all chromosomes (Figure 11a).

**Figure 12:**
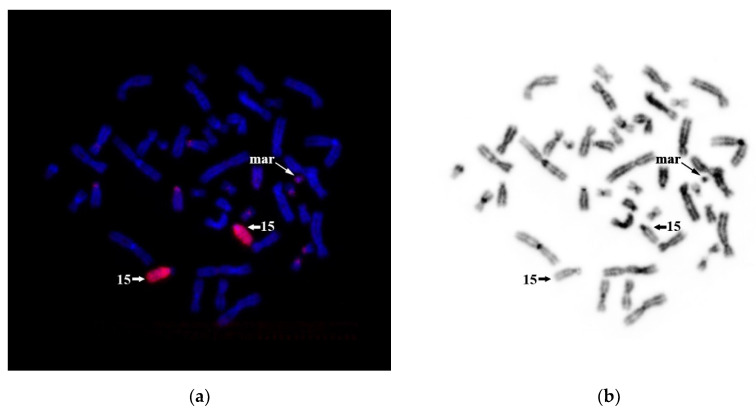
CISS hybridization with WCP15 DNA probe on the patient’s metaphase chromosomes. (**a**) WCP15 DNA probe-stained chromosome 15 (red). A weak signal in nucleolar organizer regions (NORs) of acrocentric chromosomes. (**b**) Inverted DAPI banding. No. 15—indicates the homologs of chromosome 15. mar—indicates sSMC.

Because around 80% of the sSMCs derived from acrocentric chromosomes initiate from chromosome 15 (Liehr, 2013), we tested our hypothesis of sSMC origin from HSA15 by CISS hybridization with WCP15. The suppression of hybridization of repeated sequences was designed in a way that WCP15 completely stains HSA15—its euchromatin and heterochromatin regions (Figure 12). We detected a weak signal at the short arms of other acrocentric chromosomes and a signal of a similar intensity at one of the arms of the sSMC. This signal is due to rDNA clusters and other homologous repetitive sequences at the short arms of acrocentric chromosomes and the sSMC. This result allowed us to conclude that sSMC formation is not associated with chromosome 15.

**Figure 13:**
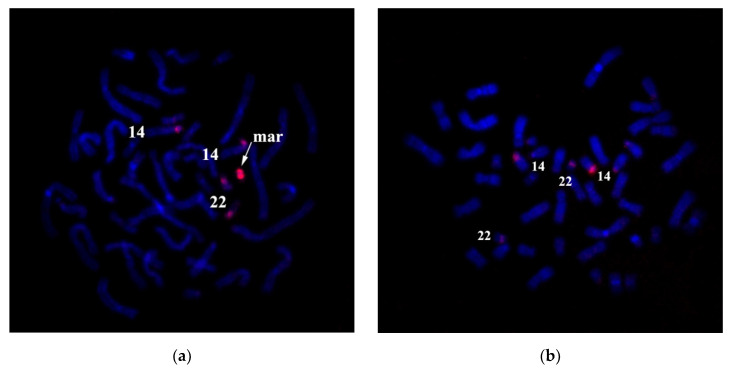
FISH of DNA probe, obtained by microdissection from one copy of a marker chromosome on metaphase chromosomes of the patient (**a**) and a healthy individual (**b**). mar and arrows indicate sSMC.

Based on the above information, we produced a DNA probe from one copy of the sSMC to determine its origin. CISS hybridization with this DNA probe labeled sSMC, and the short arms and pericentromeric regions of chromosomes 14 and 22 (Figure 9a). Pericentromeric regions of these chromosomes include a significant number of homologous sequences. Finding conditions suitable for the suppression of hybridization of homologous sequences during CISS hybridization enabled us to decrease unspecific signal intensity at the pericentromeric region of chromosome 22, preserving the specific signal intensity at chromosome 14 (Figure 13a,b).

**Figure 14:**
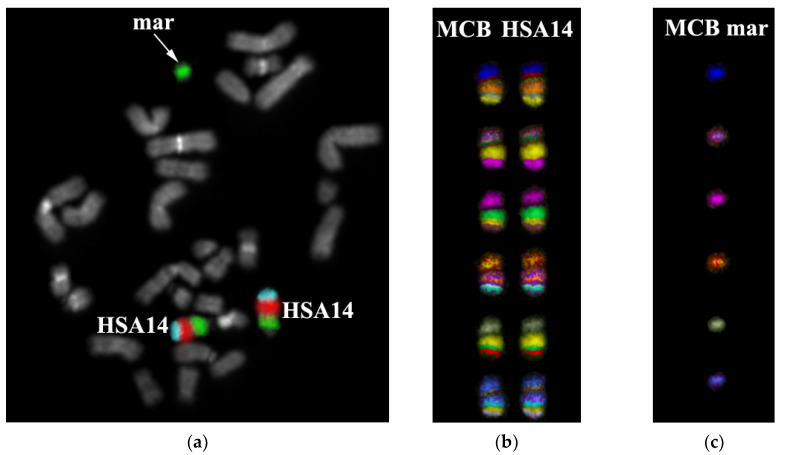
Three-color FISH with region-specific DNA-probes (PCP14a (green), PCP14b (red), PCP14c (bright blue) on the patient’s metaphase chromosomes. (**a**) Chromatin DAPI staining in blue. Inverted DAPI banding. (**b**) Patient’s chromosome 14 and (**c**) MCB of sSMCs are marked.

For the confirmation of the sSMC origin from chromosome 14, we produced several region-specific microdissectional DNA probes from chromosome 14 (14pter→p11 (PCP 14a), 14p11→q22 (PCP14b), 14q22→qter (PCP14c). Such a combination, consisting of three PCP with a respective signal level, accounts for the multicolor banding (MCB) of chromosome 14 (Figure 14). MCB allows for determining the order and the orientation of chromosomal regions. Notably, the size of regions detected by MCB is always slightly bigger than regions detected via reverse hybridization of chromosome-specific microdissectional DNA probes. To acquire the MCB, we used different classifications of pseudocolors. All of them labeled sSMC and a pericentromeric region of chromosome 14 in a similar fashion, suggesting the originating of the sSMC from chromosome 14 and its pericentromeric region.

**Figure 15:**
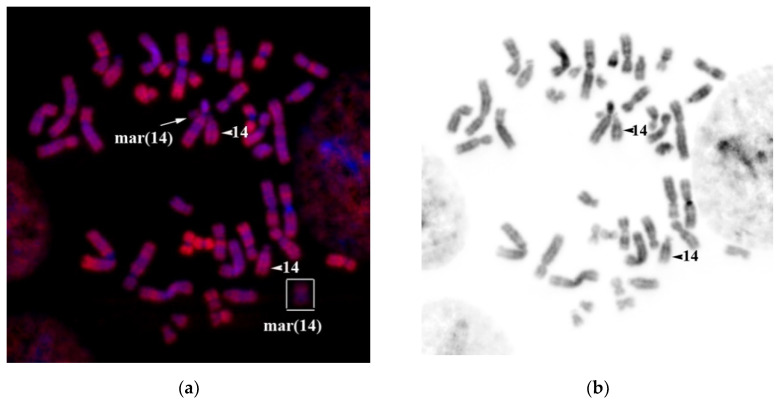
FISH (**a**) Alu-DNA probe on the patient’s metaphase chromosomes (red). On the right—marker chromosome labeled with a DNA probe detecting euchromatin. Chromosome 14 and the sSMC are indicated. (**b**) Inverted DAPI-banding. Chromatin DAPI staining is in blue. mar(14) and arrows indicate sSMC. Arrowhead indicates chromosome 14.

Obtained results enable to describe sSMC as del(14)(p13q11). A crucial aspect is to identify the localization of a breakpoint in 14q11.1 and 14q11.2. If the breakpoint is in 14q11.1, then the sSMC does not contain euchromatin material of the long arm of chromosome HSA14. In the case of a breakpoint inside 14q11.2, the genes of a sub-band 14q11.2 could potentially localize in the sSMC leading to the increase in their respective copy numbers. Sub-band 14q11.2 is G-negative suggesting its enrichment with Alu-repeats. We have performed a hybridization with a labeled Alu-DNA probe on the patient’s metaphase chromosomes to check for the presence of the 14q11.2 sub-band inside the sSMC. The intensity of the signal from the Alu-DNA probe in the sSMC was considerably lower than the intensity of a signal in G-negative regions of chromosomes and did not exceed the signal intensity in the satellites of acrocentric chromosomes containing a small number of Alu repeats (Figure 15).

Our complex molecular cytogenetic analysis of the origin and the contents of the patient’s marker chromosome shows that it is derived from chromosome 14: del(14)(p13q11.1) and does not contain transcriptionally active chromatin.

## 4. Discussion

### 4.1. Complex Chromosomal Rearrangements

In cytogenetic diagnostics, molecular cytogenomic techniques are becoming increasingly prevalent, and they do not require using a patient’s metaphase chromosomes. One of the most popular strategies to diagnose chromosomal abnormalities includes the analysis of GTG-labeled metaphase chromosomes and the consequent scrutinizing using microarray CGH or mass parallel sequencing. The current study results prove that, sometimes, to identify a chromosomal abnormality, one needs to perform CISS hybridization with whole chromosome DNA probes, which label potentially fragmented chromosomes. Producing microdissectional DNA probes from such chromosomes enables identifying chromosomal abnormalities that are not detected with cytogenomic methods. Moreover, our CCR analysis results suggest that a complex molecular analysis could detect additional chromosomal rearrangements that are critical even in the case of a preserved chromosomal region balance.

The alteration of the composition of topology-associated domains (TAD) could potentially affect the regulation of replication and transcription in a chromosomal region with a large number of genes. TAD structural analysis could be performed using Hi-C; however, it all could be much more effective knowing what exact chromosomal regions need to be analyzed thoroughly. The percentage of chromosomal abnormalities detected via molecular cytogenetic or cytogenomic analysis in patients with a normal karyotype according to GTG-differential staining but having phenotypic abnormalities is relatively low, around 5–10% [28,29]. For instance, among patients with idiopathic mental retardation (MR), developmental delay (DD), and/or nonspecific dysmorphic features (NDF) and normal karyotype, described based on GTG-differential analysis, using subtelomeric DNA probes enabled us to detect distinct chromosomal rearrangements. Their complete characterization required performing an additional FISH with chromosome-specific and locus-specific DNA probes. In our follow-up study, we detected the insertion of a small region of chromosome 3 into chromosome der(5). This enabled us to determine the sequence of events and to offer a mechanism of formation of insertion 3q25 in chromosome der(5). The formation of a final karyotype potentially proceeded as a two-step process involving multiple cell generations.

Unlike GTG banding, FISH diagnostics presented here enabled us to detect the chromosome 3 segment within the der(5) chromosome. Besides describing a translocation, which involves three chromosomes, we could also identify a small region of chromosome 3 on chromosome der(5). That was possible due to the “multiplex” application of WCPs from intact chromosomes, as well as of a WCP from rearranged chromosomes. The detection of a small region of chromosome 3 on chromosome der(5) raised additional questions about the significance of the newly identified chromosomal breakpoints, emphasizing the possible impairment of TAD structures and microdeletion formation in this region.

The detection of rearranged chromosomes is of high importance for medical genetic diagnostics and prediction. A probability of gamete formation with balanced chromosomes is extremely low in such a complex chromosomal rearrangement. The family is recommended in vitro fertilization (IVF) using donor oocytes. In the case of spontaneous pregnancy, the patient is recommended to undergo prenatal diagnostic testing.

According to the literature, many reciprocal translocation carriers are phenotypically normal, but they have an increased risk of having children with chromosomal imbalance, spontaneous abortion, or infertility [1].

### 4.2. Small Supernumerary Marker Chromosomes

Among chromosomal abnormalities, sSMC represents a special type of chromosomal rearrangement, which has been very problematic in diagnostics until recently. sSMC detection requires an analysis of the patient’s metaphase chromosomes. Cytogenomic techniques (CGH or mass parallel sequencing) can only detect an increase in copy numbers for a specific euchromatin region within sSMC. It is still a question whether it derives from sSMC or one of the chromosomes. If sSMC lacks such a euchromatin region, it escapes detection. Notably, most sSMCs are present only in some of the cells, which could also hamper or even completely block their detection via cytogenomic methods. We suggest that an optimal sSMC identification and characterization strategy is to analyze the patient’s metaphase chromosomes. The determination of its origin and contents should be performed using DNA probes specific for pericentromeric chromosomal regions or making microdissectional DNA probes from sSMC with a consequent reverse painting. These steps help to identify sSMC frequency in a patient’s cells and its origin. In the case of de novo formed sSMCs, researchers can use this information to check for monoparental disomy in the initial chromosome.

Amplifying and using microdissectional DNA probe from sSMC also enables us to determine whether it contains any euchromatin. If sSMC is present in all patient’s cells, CGH and mass parallel patients’ genome sequencing allows clarifying what euchromatin region of the initial chromosome is preserved in sSMC.

FISH results with a microdissectional DNA probe on metaphase chromosomes could prove complicated to interpret in case of a presence of only a small euchromatin region. At the same time, karyotype mosaicism could question or even exclude CGH or mass parallel sequencing applicability. In such a scenario, FISH with labeled Alu-repeat helps to detect euchromatin material within sSMC more reliably than FISH with microdissectional DNA probe [30]. Another approach to describe sSMC is to sequence the microdissectional DNA library derived from the sSMC. This enables us to detect euchromatin’s presence within sSMC and rather accurately describe its contents [31,32]. However, this approach’s application demands longer periods, which conflicts with the short turnaround time required for prenatal diagnostic testing.

Unfortunately, even a detailed description of the sSMC contents does not solve all medical genetic counseling problems. A complete absence of euchromatin within the sSMC suggests that the sSMC itself could not lead to the formation of an abnormal phenotype. However, in the case of its de novo formation, one must perform an additional test for UPD in the initial chromosome. It has also proved complicated to assess the pathogenic capabilities of the sSMC, which contains euchromatin, except the sSMCs derived from chromosome 15. Identifying the most frequent locations of chromosomal breakpoints during the formation of such sSMC and many studied cases allows for reliably assessing its clinical implications and consequences. If sSMCs are derived from other chromosomes, such an assessment becomes more problematic.

Multiple studies show that sSMCs, in which the size of a euchromatin region does not exceed 3–5 megabase pairs, do not possess any pathogenic potential [33]. Nevertheless, a work by Marle has demonstrated that the risk of an abnormal phenotype could be associated with the size of the euchromatin region (more or less than 1 Mb) or the number of genes (more or less than 10) [34]—these studies need to be continued. The underlined characteristic of sSMCs with small euchromatin regions could potentially be associated with its localization in the interphase nucleus. A small euchromatin material and pericentromeric sSMC heterochromatin could be placed into the peripheric transcriptionally inactive nuclear compartment leading to the suppression of the transcriptional activity. To avoid such suppression, the euchromatin region must potentially exceed a specific size. Indeed, the analysis of B chromosome spatial organization in association with heterochromatin regions of autosomes in Korean field mice Apodemus peninsulae matches well with the above hypothesis [32,35]. On the contrary, in birds, microchromosomes are characterized by high transcriptional activity and are in the inner transcriptionally active nuclear compartment [36].

Concluding, one must mention that besides the high effectiveness and broad capacities of modern cytogenomic methods, performing a well-rounded, complete diagnostic of chromosomal abnormalities requires complex studies utilizing both the cytogenomic techniques as well as traditional cytogenetic methods. Interestingly, copy number variation (CNV) in different sizes is rather frequently detected by CGH, but the interpretation of the results is often problematic. In the current paper, we describe the structural changes in chromosomes, which were detected or expected after GTG banding. The sSMC lacks euchromatin, suggesting that its formation does not lead to the imbalance of the genome’s euchromatin portion. Notably, de novo formed sSMCs are associated with a high risk of uniparental disomy, therefore requiring performing additional tests. Analysis of the complex chromosomal rearrangement also fits well with the notion that it does not cause genetic imbalance. However, identifying the points of breaks and reassociations during the chromosomal rearrangements allows for precisely monitoring these regions, which could be further scrutinized using diverse molecular diagnostic techniques. Significantly, such chromosomal rearrangements cause serious reproductive issues while not leading to genetic imbalance.

## 5. Conclusions

Application of WCPs derived from metaphase chromosomes for the painting of rearranged ones allows the detailed description of their content. In the present study, the approach included applying WCPs derived from abnormal chromosomes; it appeared to be efficient in molecular cytogenetic diagnostics of chromosomes formed with some subsequent translocations and small supernumerary marker chromosomes. CISS hybridization with WCP derived from abnormal chromosomes can precisely define its content, while CISS hybridization with WCPs from intact chromosomes can confirm a conclusion based on a study using WCP generated from the chromosome of interest. Results obtained in the performed analysis of the complex chromosomal abnormality and small supernumerary chromosome showed high efficiency of combining the usage of both types of WCPs and locus-specific probes with studying abnormal karyotypes revealed with the traditional cytogenetic technique of metaphase chromosome GTG-banding in routine karyotype diagnostics.

## Supplementary Materials

The following are available online at https://www.mdpi.com/2073-4425/11/12/1511/s1, Figure S1: FISH analysis of a complex chromosomal rearrangement involving three chromosomes and small supernumerary marker chromosome in patients with impaired reproductive function.
