# Supplementary material for: Two Separate Cases: Complex Chromosomal Abnormality Involving Three Chromosomes and Small Supernumerary Marker Chromosome in Patients with Impaired Reproductive Function
Source: Genes (Basel). 2020 Dec 17;11(12):1511. doi: 10.3390/genes11121511 (PMC7766715; doi:10.3390/genes11121511)
Supplement: Supplementary file 1 [file genes-11-01511-s001.pdf]

FISH analysis of a complex chromosomal rearrangement involving three chromosomes and small supernumerary marker chromosome in patients with impaired reproductive function

Method of analysis

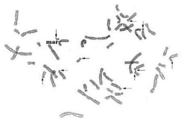

GTG - banding

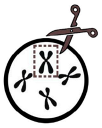

Production of DNA probes via microdissection an consequent amplification of dissected material by DOP-PCR

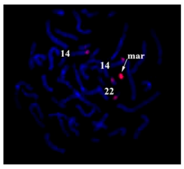

FISH of microdissection DNA probes on patients' metaphase chromosomes*.

Note. If a DNA probe is derived from an abnormal chromosome, the FISH is performed on metaphase chromosomes of both the patient and a healthy volunteer. FISH with a loci specific DNA probes have been used to identify regions in rearranged chromosomes.

Described cases

Initial description of the patient's karyotype	Results of FISH analysis
47,XY,+mar	47, XY, + del(14)(p13q11.1)
46,XX,t(3;16;5)(q25;q21;p15.1)	46,XX,der(3)(3pter→3q25::5p15.1→5pter),der(5)(16qter→16q21::3q25::5p15.1→5qter),der(16)(16pter→16q21::3q25→3qter)

Figure S1. FISH analysis of a complex chromosomal rearrangement involving three chromosomes and small supernumerary marker chromosome in patients with impaired reproductive function.
